# A Subset of Osteoblasts Expressing High Endogenous Levels of PPARγ Switches Fate to Adipocytes in the Rat Calvaria Cell Culture Model

**DOI:** 10.1371/journal.pone.0011782

**Published:** 2010-07-26

**Authors:** Yuji Yoshiko, Kiyoshi Oizumi, Takuro Hasegawa, Tomoko Minamizaki, Kazuo Tanne, Norihiko Maeda, Jane E. Aubin

**Affiliations:** 1 Department of Oral Growth and Developmental Biology, Hiroshima University Graduate School of Biomedical Sciences, Hiroshima, Japan; 2 Biological Research Laboratories III, Daiichi Sankyo Co., Tokyo, Japan; 3 Department of Orthodontics and Craniofacial Developmental Biology, Hiroshima University Graduate School of Biomedical Sciences, Hiroshima, Japan; 4 Department of Molecular Genetics, Faculty of Medicine, University of Toronto, Toronto, Ontario, Canada; National University of Singapore, Singapore

## Abstract

**Background:**

Understanding fate choice and fate switching between the osteoblast lineage (ObL) and adipocyte lineage (AdL) is important to understand both the developmental inter-relationships between osteoblasts and adipocytes and the impact of changes in fate allocation between the two lineages in normal aging and certain diseases. The goal of this study was to determine when during lineage progression ObL cells are susceptible to an AdL fate switch by activation of endogenous peroxisome proliferator-activated receptor (PPAR)γ.

**Methodology/Principal Findings:**

Multiple rat calvaria cells within the ObL developmental hierarchy were isolated by either fractionation on the basis of expression of alkaline phosphatase or retrospective identification of single cell-derived colonies, and treated with BRL-49653 (BRL), a synthetic ligand for PPARγ. About 30% of the total single cell-derived colonies expressed adipogenic potential (defined cytochemically) when BRL was present. Profiling of ObL and AdL markers by qRT-PCR on amplified cRNA from over 160 colonies revealed that BRL-dependent adipogenic potential correlated with endogenous PPARγ mRNA levels. Unexpectedly, a significant subset of relatively mature ObL cells exhibited osteo-adipogenic bipotentiality. Western blotting and immunocytochemistry confirmed that ObL cells co-expressed multiple mesenchymal lineage determinants (runt-related transcription factor 2 (Runx2), PPARγ, Sox9 and MyoD which localized in the cytoplasm initially, and only Runx2 translocated to the nucleus during ObL progression. Notably, however, some cells exhibited both PPARγ and Runx2 nuclear labeling with concomitant upregulation of expression of their target genes with BRL treatment.

**Conclusions/Significance:**

We conclude that not only immature but a subset of relatively mature ObL cells characterized by relatively high levels of endogenous PPARγ expression can be switched to the AdL. The fact that some ObL cells maintain capacity for adipogenic fate selection even at relatively mature developmental stages implies an unexpected plasticity with important implications in normal and pathological bone development.

## Introduction

Multipotent mesenchymal stem cells differentiate into osteoblasts, adipocytes and other mesenchymal lineages, and key transcription factors underlie commitment and fate choices of cells to particular lineages with suppression of alternative lineages [1], [2], [3]. Considerable evidence supports the notion that osteoblasts and adipocytes are closely related through a common progenitor. For example, a decrease in bone volume in age-related and steroid-induced osteoporosis is accompanied by an increase in marrow adipose tissue (see for example, [4], [5]). A variety of experimental manipulations in primary bone marrow stromal cells and cell lines have contributed molecular and cellular insight into the mechanisms underlying the apparent reciprocal relationship between the two lineages (see for example, [1], [6], [7], [8], [9], [10], [11]). These studies have led to the suggestion that regulated lineage allocation of stem or multipotential progenitor cells or a fate switch from osteoblast lineage (ObL) to adipocyte lineage (AdL) occurs under certain conditions, including aging. However, it is unclear at what commitment or differentiation stage(s) fate changes occur.

Peroxisome-proliferator activated receptor (PPAR)γ, a ligand-activated transcription factor belonging to the nuclear hormone receptor superfamily, is expressed principally in adipose tissue and heterodimerizes with a retinoid X receptor to bind the PPAR response elements within the promoters of target genes, including adipocyte-associated genes. Thus, PPARγ (PPARγ2, in particular) acts as a master regulator of the adipocyte developmental program together with other transcription factors, such as PPARα and CCAAT enhancer-binding proteins (C/EBPs) [12], [13], [14]. Thiazolidinediones, anti-diabetic agents including rosiglitazone (BRL-49653 (BRL)), are frequently used synthetic ligands for PPARγ [15] and stimulate adipogenesis and inhibits osteoblastogenesis in vivo and in vitro [8], [9], [10], [11]. This may be implicated in downregulation of runt-related transcription factor 2 (Runx2) in a bone marrow-derived cell line overexpressing PPARγ2 [16].

Evidence from PPARγ haploinsufficient mice also supports the concept that PPARγ is antagonistic to osteogenesis, acting early in mesenchymal cell differentiation [1]. In contrast, microarray analysis of mesenchymal lineage markers in mouse calvaria cell cultures indicates that adipocyte- (except PPARγ) but not myocyte-asssociated genes are transcriptionally induced together with osteoblast-associate genes during osteoblast development [17]. Similarly, our recent data show that adipocytes emerge along with osteogenic potential in a fraction of fetal rat calvaria cells treated with BRL [18]. Developmental regulation of calvaria cells, a frequently used model of ObL cells, is different in at least certain respects, from that of marrow stromal cells, suggesting that these diverse results may reflect differences in PPARγ regulation of osteo-adipogenic fate choice in the two organs. However, dissecting when during ObL lineage progression cells may be susceptible to fate switches is complicated by the fact that although osteoblast differentiation is well characterized [19], phenotypic heterogeneity of ObL cells is seen in multiple cell culture models, including stromal and calvarial cells, as well as in developing rat calvariae [20]. This heterogeneity includes cellular responses to cytokines and hormones (e.g., parathyroid hormone (PTH)/PTH related protein receptor in cortical versus trabecular bone compartments [21]).

Based on all of these data, we hypothesized that subsets of ObL cells may differentially respond to PPARγ. By preparing ObL cells at multiple differentiation stages from rat calvariae either by magnetic cell sorting using anti-alkaline phosphatase (ALP, a marker of relatively early ObL progression) antibody or by replica plating of single cell-derived colonies, we identified and characterized a distinct subset of ObL cells that can convert into adipocytes in the presence of BRL.

## Results

### Multiple cellular pathways lead to adipogenesis in ObL cells

**Figure 1:**
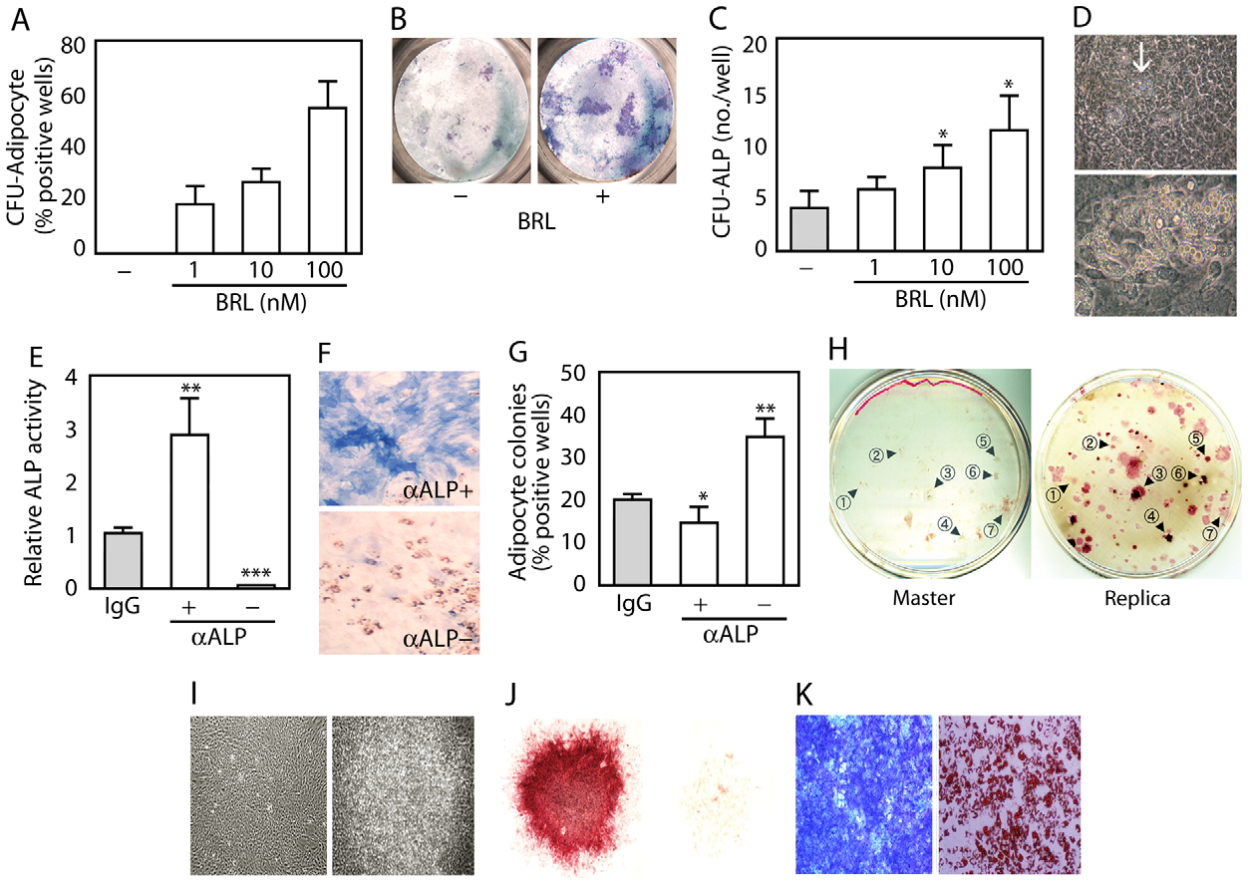
BRL induces adipocytes in ObL cells. (A–C) RC cells in 96-well plates (150 cells/well) were cultured in osteogenic medium with or without 1–100 nM BRL for 10 days. (A) Percentage of wells containing CFU-adipocyte. (B) Representative stereoscopic images of CFU-ALP colonies. (C) Number of CFU-ALP per well. n = 32 wells/treatment. *p<0.05, compared to control (vehicle alone). (D) Adipocytes within mineralized bone nodules. Cells were treated with 100 nM BRL for 5 days after mineralized bone nodules had formed (day 14). Phase-contrast microscopic images; lower panel, the enlargement of adipocytes (arrow) in upper panel. (E–G) RC cells at day 10 were fractionated by magnetic cell sorting with anti-ALP antibody (αALP) or normal IgG (control). (E) Quantification of ALP activity in ALP^+^, ALP^−^ and control fractions. (F) ALP (blue)/oil red O (red)-double staining in ALP^+^ (αALP+) and ALP^−^ (αALP-) fractions shown by stereoscopy. The remainder of each fraction in (E) was cultured in osteogenic medium with 100 nM BRL for a week. (G) Percentage of wells containing adipocyte colonies in the ALP^+^, ALP^−^ and control fractions. n = 16 wells/fraction. ***p<0.001, **p<0.01 and *p<0.05, compared to control (IgG). (H–K) RC cells at very low density (≤15 cells/cm^2^ in 100-mm dishes) in osteogenic medium plus 10 nM Dex (master dishes). A few days later, polyester cloths were overlaid on master dishes for a day to make replicas. 10 mM βGP was added into replica dishes. (H) Master and replica dishes. Master and replica dishes (day 25) were fixed and stained with hematoxylin and ALP/von Kossa, respectively. Arrowheads and numbers in each dish indicate examples of matched pairs of colonies. (I) Phase-contrast microscopy of single cell-derived colonies in the master dish on day 17. Left and right panels show typical monolayer and multilayer colonies, respectively. (J) ALP^+^ (left) and ALP^−^ (right) colonies in the master dish on day 15. (K) Osteo-adipogenic potential in osteoblast-lineage colonies. Subcultures from individual colonies were maintained for a week in osteogenic medium with 100 nM BRL. Left panel, ALP staining of cells from a colony on day 21. Right panel, oil red O staining of cells from a colony on day 12.

As described previously [18], ObL cells from fetal rat calvariae proliferated (day 0–5), reached confluence and subsequently formed cell condensations (day 5–6), followed by nodule formation (differentiation, day 6–10) (Fig. S1) and mineralization (maturation, not shown). This developmental process was also confirmed by gene expression profiling of osteoblast markers (Fig. S2A). ObL cells chronically treated with BRL were morphologically similar to those without BRL up to day 5–6, and thereafter adipocytes were seen within cell condensations (Fig. S1). In contrast to what was seen in BRL-treated cultures, a very few adipocytes were dispersed in control cultures without BRL, but they disappeared as ObL progression occurred (data not shown). Likewise, colony formation assays revealed that there were only a few colony-forming unit (CFU)-adipocyte colonies without BRL (Fig. 1A). BRL, however, increased not only CFU-adipocyte (Fig. 1A) but also CFU-ALP colonies (Fig. 1B,C). When we treated cells in which mineralized nodules had already formed with BRL for 5 days, a few adipocytes were present within some of the mineralized nodules (Fig. 1D), raising the possibility that adipocytes may arise even from relatively mature ObL cells. To separate mature ObL cells from multipotential progenitors (e.g., side population cells [22]), and other potentially contaminating lineages present in the primary cell digestion, we next released cells by enzymatic digestion from nodule-forming cells (day 10), and separated them into ALP-positive (ALP^+^ **)** and ALP-negative (ALP^−^) fractions [23] by magnetic cell sorting (Fig. 1E). Adipocytes formed diffusely (Fig. 1F) in all fractions treated with BRL, although the number of adipocytes present varied with ALP^−^>control/unfractionated>ALP^+^ (Fig. 1G).

To define whether adipogenic potential is restricted to a specific subset of ObL cells, we used a combination of single cell colony assays and replica plating [24], [25]. ObL colonies were retrospectively identified by ALP/von Kossa staining of their corresponding replicas (Fig. 1H). Cells in individual colonies displayed homogenous morphology (Fig. 1I) and ALP activity, i.e., colonies comprised ∼100% ALP^−^ or ALP^+^ cells (Fig. 1J). Over 160 colonies from 12, 15, 17, and 21 days of culture were collected in each of two independent experiments; a portion of cells of each colony was re-plated at high cell density with BRL, while the remainder was collected for total RNA preparation. One hundred-fifteen and 132 colonies in each experiment were successfully adapted to subculture, and of these, 94 and 95 respectively were designated osteoblast lineages, as verified by replica dishes (Table 1).

**Table 1:** Summary of colony types and developmental fate of single cell-derived rat calvaria cell colonies.

Colony types	Number of colonies							
	Experiment 1				Experiment 2			
(Days)	12	15	17	21	12	15	17	21
Total, recovered from master dishes	51 (18)	34 (30)	42 (34)	41 (39)	55 (11)	39 (32)	38 (35)	40 (38)
Total, subcultured successfully	26 (17)	29 (26)	34 (26)	28 (28)	33 (8)	31 (23)	30 (28)	38 (36)
ALP positive	1 (1)	3 (3)	7 (7)	21 (21)	3 (2)	5 (5)	7 (7)	28 (28)
Oil red O positive	16 (14)	3 (3)	2 (2)	0 (0)	11 (4)	3 (2)	6 (5)	0 (0)
Double positive	2 (2)	14 (14)	11 (11)	3 (3)	1 (1)	14 (13)	10 (10)	2 (2)
Double negatve	7 (0)	9 (6)	14 (6)	4 (4)	8 (1)	9 (3)	7 (6)	8 (6)

Numbers in parentheses indicate definitive osteoblast-lineage colonies retrospectively-identified by replica plating.

Definitive ObL colonies subcultured and treated with BRL were classified into four categories based on ALP and oil red O staining: single ALP^+^ is defined as osteogenic (32/97 and 42/95 in experiment 1 and 2 respectively); oil red O^+^ is adipogenic (19/97 and 11/95); double positive ALP^+^/oil red O^+^ are osteo-adipogenic colonies (30/97 and 26/95), and double negative ALP^−^/oil red O^−^ are neither osteo- nor adipogenic (16/97 and 16/95) (Table 1 and Fig. 1K). Notably, colonies picked and subcultured at day 12 exhibited mainly monopotential adipogenic fate at subculture, while colonies picked on day 21 were mainly monopotential but for osteogenic fate. Double-positive colonies/bipotential fate occurred mainly in colonies isolated at days 15, 17 and 21. Taken together, the results suggest that some ObL cells, including cells already partially differentiated/maturing, can adopt an adipo/osteogenic fate, but that mature osteoblasts have a much lower probability to do so, at least under the conditions tested.

### Gene expression profiling of single cell-derived colonies and its relationship to osteo/adipogenic potential

**Figure 2:**
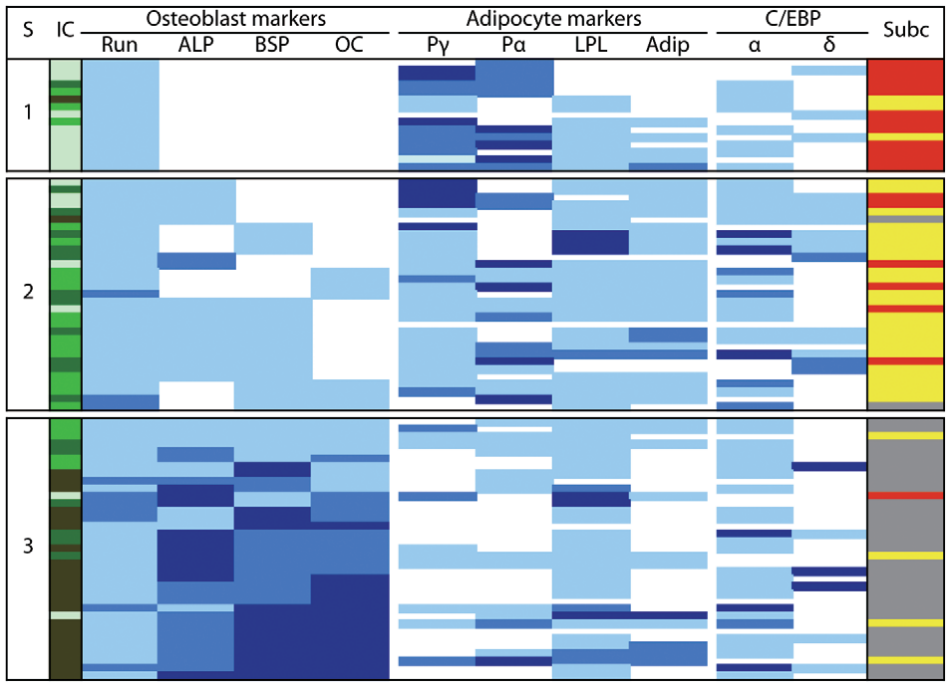
Gene expression profling of osteoblast/adipocyte markers in single cell-derived ObL colonies and their osteo-adipogenic potential. Numbers in each column denote relative mRNA levels of osteoblast- and adipocyte-related markers by qRT-PCR. Light blue, blue and dark blue denote categories of expression levels, and are relatively low (1.0–1.9), intermediate (2.0–2.9) and high (≥3.0), respectively. Blank space, Undetectable. S, Stage-1, immature; 2, intermediate; 3, mature, according to expression profiling of osteoblast markers. IC, Individual colonies. Colors in IC imply colonies derived from the same culture days (light green, day 12; green, day 15; dark green, day 17; olive, day 21). Run, Runx2; OC, OCN; Pγ and Pα, PPARγ and PPARα, respectively; Adip, Adipsin. Subc, Colonies subcultured with BRL. Red, Oil red O positive; Gray, ALP positive; Yellow, Oil red O/ALP double positive.

We next used real-time quantitative RT-PCR (qRT-PCR) to analyze expression of osteo-adipogenic markers and transcription factors necessary for mesenchymal lineage progression in representative colonies (97 ObL colonies subcultured in experiment 1, Table S1). Based on their osteoblast marker expression and the established osteoblast hierarchy [19], colonies were rearranged into an order from early (immature) to late stages of ObL progression, i.e., immature (negative for all of ALP, bone sialoprotein (BSP) and osteocalcin (OCN)), intermediate (negative for either ALP, BSP, or OCN) or mature (positive for all osteoblast markers) colonies (Fig. 2). It is important to note that all colonies listed were committed to the ObL, as evidenced by Runx2 gene expression and ALP/von Kossa staining outcomes in replica dishes as described above. About 20% of colonies in subcultures supplemented with BRL were double negative (Table 1) and had fibroblastic morphology (not shown). There was a clear developmental stage dependency in the frequency with which osteo-adipogenic mono- or bipotential colonies occurred; analysis by expression profiling was similar to, but more robust than, what was detected by cytochemistry (Table S1 and Fig. 2). There were significant differences in PPARα and PPARγ mRNA levels in colonies that gave rise to either ALP^+^ or oil red O^+^ and between ALP^+^/oil red O^+^ and oil red O^+^ colonies in BRL-treated matched subcultures (Table 2). Further, PPARα mRNA levels in colonies monopotential for adipogenic fate (oil red O^+^) were significantly higher than those in colonies defined as osteo-adipogenic bipotential (Table 2). In contrast, C/EBPs expression levels and osteo-adipogenic activities were poorly or not correlated (Table 2). Taken together, the data suggest that the adipogenic potential of ObL cells may be defined by the relative levels of PPARγ and PPARα versus other marker mRNAs.

**Table 2:** Expression levels of PPAR and C/EBP mRNAs in ObL colonies correlates with their osteo-adipogenic potential when subcultured in the presence of BRL.

Number of colonies		19	30	32
Staining pattern in subcultures		ORO	ORO/ALP	ALP
Relative mRNA levels	PPARγ	2.22±1.18^a^	1.82±0.95^a^	0.24±0.58
	PPARα	2.18±1.22^a^	1.01±1.22^b,^ c	0.35±0.53
	C/EBPα	0.71±0.78	1.12±1.06	1.12±0.84
	C/EBPδ	0.21±0.42	0.47±0.71	0.44±0.98

ORO, Oil red O positive; ALP, ALP positive; ORO/ALP, Oil red O/ALP double positive in subcultures with BRL.

a p<0.001 and ^b^p<0.01, compared to matched ALP.

c p<0.05, compared to matched.

**Figure 3:**
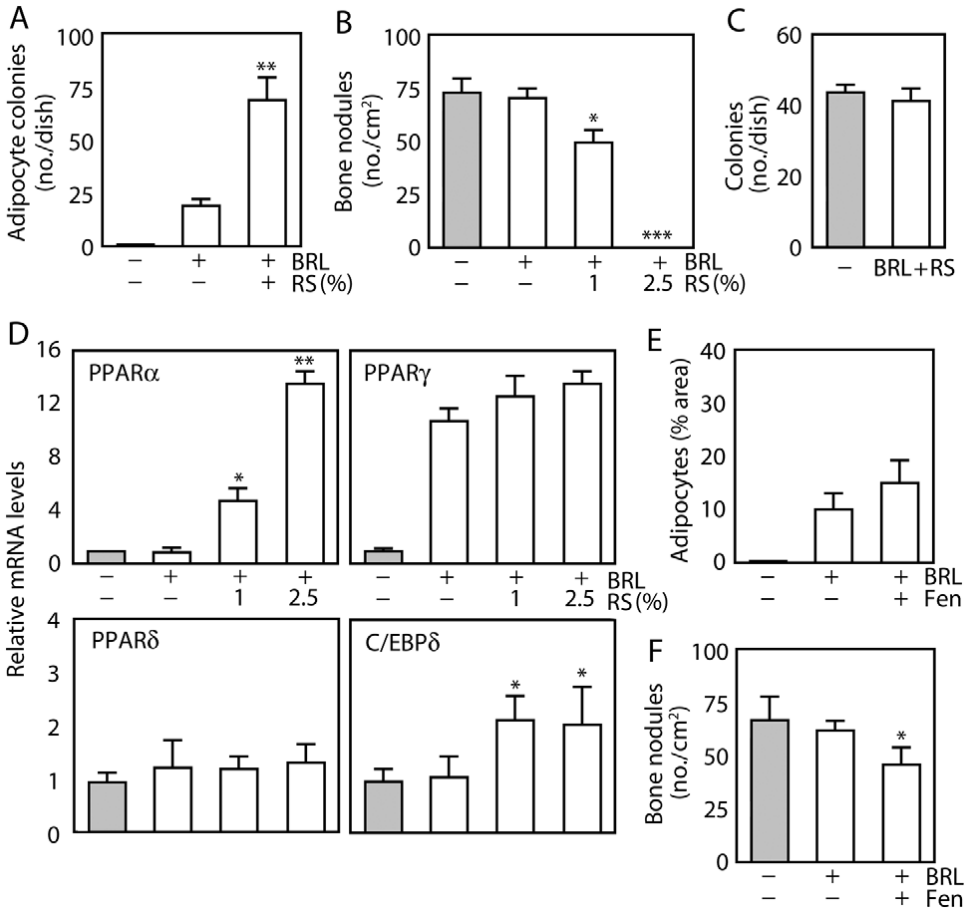
RS modifies the BRL effects in RC cell population cultures. Cells in osteogenic medium were chronically treated with or without BRL in combination with or without RS or fenofibrate for 14 days. (A and B) A combination of 100 nM BRL and 1–2.5% RS elicits a reciprocal increase in adipocytes (A) and decrease in bone nodules (B). Cells were double stained with ALP and oil red O. (C) RS does not change the total colony number in BRL/RS-treated cultures. Cells were plated at very low density as described in Fig. 1 and maintained for 21 days with or without 100 nM BRL plus 2.5% RS. (D) RS increases the mRNA levels of PPARα and C/EBPδ but not PPARγ and PPARδ. Total RNA was isolated from cultures on day 14, and qRT-PCR was performed. (E and F) Fenofibrate (Fen, 100 nM) does not substitute for RS in BRL-treated cells. Fenofibrate (100 nM) did not completely mimic the RS effect on adipocyte colony (E) and bone nodule (F) formation. ***p<0.001, **p<0.01 and *p<0.05, compared to BRL alone.

To confirm that adipogenic fate of ObL cells is defined by relative levels of PPARγ and PPARα, we co-treated rat calvaria cells with BRL plus rabbit serum (RS), the latter known to stimulate PPARα gene expression [26]; co-treatment resulted in an inverse relationship between the number of bone nodules (decrease) and adipocyte colonies (increase) that formed (Fig. 3A,B) with no effect on total colony number (Fig. 3C). Amongst PPAR and C/EBP family members, PPARα and C/EBPδ mRNA levels were increased by RS (Fig. 3D). However, fenofibrate, a synthetic PPARα ligand [27], combined with BRL, did not fully mimic the RS plus BRL effect (Fig. 3E,F), suggesting that RS may elicit other activities, including induction of C/EBPδ, to induce adipogenesis. In any case, our results indicate that committed ObL cells unambiguously defined by marker expression profile and functional endpoints exhibit diverse molecular phenotypes as characterized by expression of non-osteoblastic mesenchymal lineage markers. That the diversity extends beyond adipogenic regulatory genes was confirmed by profiling two transcription factors involved in myogenesis (MyoD) and chondrogenesis (Sox9); these were also expressed in 22 and 20% of osteogenic colonies in developmentally immature (stage 1) and intermediate (stage 2) stages respectively (one colony expressed both), but not in any colonies at mature stages (Fig. S3).

### Individual ObL cells identical in osteoblast development stage are molecularly and functionally distinct

By assessing single cell-derived colonies, we showed that rat calvaria cells comprise a heterogeneous mixture of ObL cells with different gene expression profiles and different potential for fate switching. However, as is well-established [19], the rat calvaria cell population expresses a temporally reproducible sequence of osteoblast development (see description above and the sequential upregulation of oteopontin (OPN), ALP and OCN in Fig. S2A). We therefore also assessed mRNA expression of mesenchymal lineage-commitment transcription factors, such as Runx2, PPARγ, Sox9 and MyoD in this model (Fig. S2A). In contrast to Runx2, which increased slightly during the differentiation time course, Sox9 and MyoD were highest early (day 3) and progressively decreased thereafter. Levels of PPARγ1 and γ2 peaked at day 6 and subsequently decreased. Western blot analysis confirmed that Runx2 and PPARγ protein expression paralleled that of their mRNAs (Fig. S2B).

**Figure 4:**
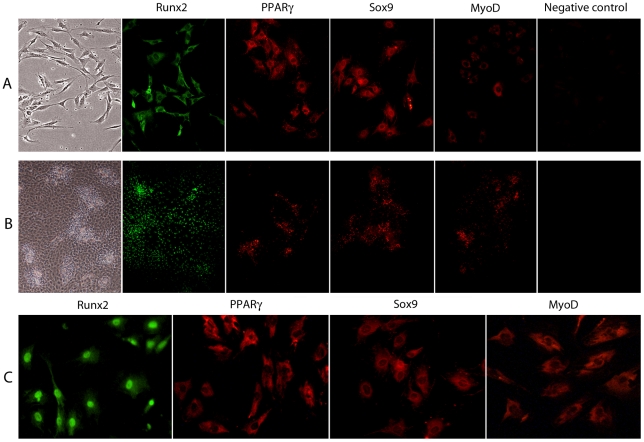
Subcellular distribution of mesenchymal lineage determinants in RC cells. Cells were cultured without BRL as described in Fig. 1. Proliferating cells (day3) (A), differentiating/nodule-forming cells (day 12) (B), and subcultures of differentiating/nodule-forming cells, i.e., cells from ALP^+^ fractions in developing RC cells (day 10) (C) were subjected to immunofluorescence staining for Runx2, PPARγ, Sox9, and MyoD. Left-side panels of A and B show proliferating and nodule-forming cells, respectively, by phase-contrast microscopy.

**Figure 5:**
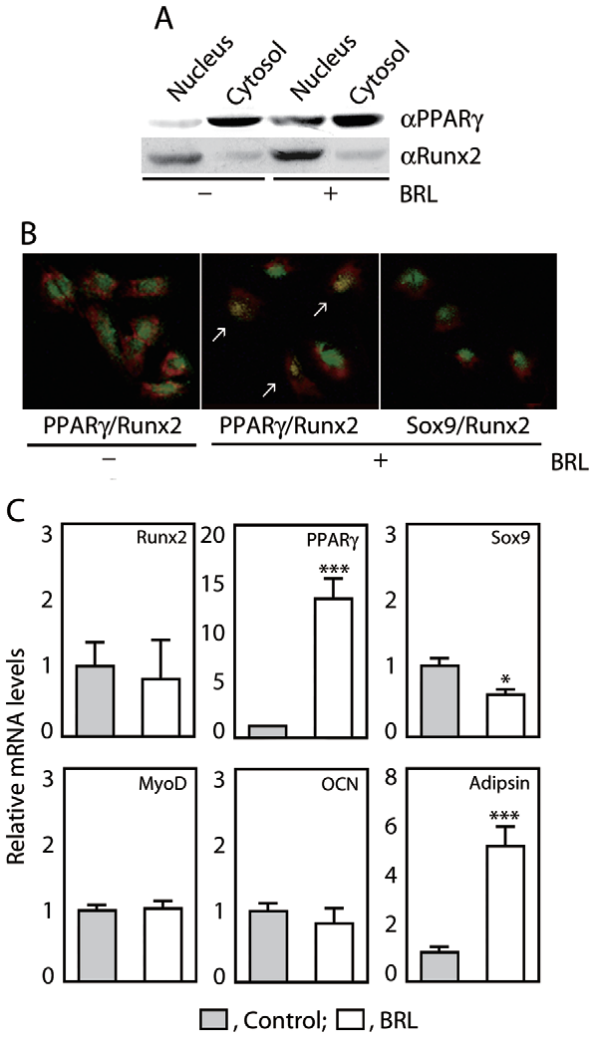
BRL promotes PPARγ actions in osteoblast-lineage cells. ALP^+^ fractions isolated from developing RC cells (day10) were subcultured with or without 100 nM BRL as described in Fig. 1E-G. (A) BRL increases relative abundance of PPARγ in the nucleus versus the cytoplasm. Cells at confluence were treated with or without 100 nM BRL for 12 h, and subcellular fractionation was performed. The nuclear and cytosolic fractions were subjected to Western blotting for PPARγ and Runx2. (B) Double PPARγ and Runx2 nuclear positive cells are present in cells cultured with BRL. After 2 days of plating, cells were treated with or without 100 nM BRL for 12 h, fixed and double stained with αRunx2 (green) and either αPPARγ or αSox9 (red). (C) PPARγ and adipsin mRNA levels are selectively increased by BRL. Cells at confluence were treated with or without 100 nM BRL for 24 h. Total RNA was extracted, and qRT-PCR for genes tested was carried out as shown in Fig. 3D.

Immunofluorescence staining of proliferating rat calvaria cells (day 3) with antibodies against the same transcription factors revealed that all were localized in the cytoplasm (Fig. 4A). All four factors were also present in cells in nodules, however the number of cells with detectable expression of PPARγ, Sox9 and MyoD in nodules was fewer than those expressing Runx2 (Fig. 4B). In subcultures of more mature cells from nodules (see above, ALP^+^ fractions), Runx2 was clearly located in the nucleus, but other factors remained cytoplasmic (Fig. 4C). Western blotting of subcultures of the ALP^+^ fraction revealed that the subcellular localization of Runx2 did not differ between cells treated or not with BRL for 12 h, whereas PPARγ was primarily localized in the nucleus in the presence but not absence of BRL (Fig. 5A). Consistent with this, some cells within the ALP^+^ fraction and positive for nuclear Runx2 were also positive for nuclear PPARγ but not for Sox9 (Fig. 5B). In parallel cultures, BRL increased PPARγ and adipsin, but did not alter mRNA levels of other transcription factors or OCN by 24 h post-induction (Fig. 5C-H). These results suggest that some ObL cells express PPARγ that remains in the cytoplasm, while others can mobilize PPARγ to the nucleus; it is presumably these latter cells that convert into adipocytes under the stimulus of PPARγ-specific ligands.

## Discussion

A comprehensive in vitro analysis of the effects of BRL on osteo-adipogenic potential in the ALP^+^ fraction of rat calvaria cells and in retrospectively identified single cell-derived definitive ObL colonies from rat calvaria cells suggests that developmentally-regulated endogenous levels of PPARs contribute to the potential of ObL cells to convert to adipocytes in the presence of BRL. We also suggest that ObL cells are heterogeneous with respect to expression of non-osteoblastic phenotypic traits, and capacity for alternative fate choices with at least some maintaining capacity for fate switches even at relatively late osteoblast differentiation stages.

As described [1], [6], [7], [8], [9], [11], bone marrow cell models in vivo and in vitro including ours [18], all support a model in which adipocytes form at the expense of osteoblasts, which may be processed at least in part by the PPARγ-mediated downregulation of Runx2 [16], [28]. Only a few studies have addressed adipogenic potential in the calvaria cell model. Runx2-deficient calvaria cells show adipo-chondrogenic bipotentiality [29], and the mouse calvaria-derived cell line MC3T3-E1 displays increased adipogenesis and decreased osteoblastogenesis with retroviral overexpression of PPARγ [30]. However, because the cells in both these latter models had lost the typical osteoblastic features before analysis, it is difficult to define unambiguously which particular ObL cells undergo transdifferentiation into adipocytes. Sorting ObL cells based on expression of ALP, a well-established osteoblast marker, allows enrichment for immature versus more mature osteoblastic precursors [23]. Thus, BRL-dependent adipogenesis in the ALP^+^ fraction of ObL cells suggests that not only immature but also maturing osteoblastic cells exhibit adipogenic potential. A combination of limiting dilution single colony assays and replica plating is a useful technique to trap cells at definable developmental stages [24], [25]. In spite of the possibility of a gradient in the proliferation-differentiation sequence from the periphery to the center of developing bone colonies [31], the uniform ALP staining we saw in colonies selected here and the lack of a relationship between the colony size (not shown) and their developmental stage suggest that a development gradient, if present under our culture conditions, was restricted to a narrow range. Using this approach, we found a statistically significant difference in the adipogenic potential amongst colonies defined as being at three development stages: immature, intermediate and mature, but adipocytes were found in all three.

Ectopic overexpression of PPARγ [30] or treatment with high doses of PPARγ ligands [8], [9], [10], [11], [16] in several models elicits reciprocal up- and downregulation of adipogenesis and osteoblastogenesis, respectively. On the other hand, BRL at relatively low concentrations has no pro-adipogenic effect in MC3T3-E1 cells [32]. Because high concentrations of PPARγ and/or their ligands may abrogate their specificity on downstream target genes [33], we used BRL at a maximum concentration of 100 nM. Indeed, we found that BRL (≤100 nM) does not induce adipocytes in MC3T3-E1 cells without PPARγ overexpression (data not shown), in agreement with previous data [32]. We also showed previously a clear difference in the capacity of BRL to alter the fate choice of precursor cells in stromal (bone marrow) versus calvaria-derived cell populations [18], and now characterize the distinct subset of ObL cells having adipogenic potential.

Fatty acid-rich RS increases PPARγ and PPARα mRNA expression in various osteoblastic cell lines (MB1.8, ROS17/2.8 and SaOS-2/B10) with a resultant decrease in ALP activity and increase in adipocyte number [26]. Likewise, we found that RS in combination with BRL mimicked the reciprocal effect of BRL on osteogenesis versus adipogenesis in bone marrow cells. However, the effect appears not to be mediated solely by PPARα and γ, because the PPARα activator fenofibrate was unable to substitute completely for RS. It is also worth noting that different PPARγ ligands have differential effects on osteo-adipogenesis in vitro and in vivo and that the adipogenic and anti-osteogenic processes can be regulated independently [9], [34], [35]. For example, additional factors such as the basic-leucine zipper class of transcription factors, C/EBPα, β and δ, are known to participate in adipogenesis [14]. Ectopic co-overexpression of C/EBPα and PPARγ induces adipogenesis in the G8 myoblast cell line [36] as well as in MC3T3-E1 cells [30]. Although it was fairly widely expressed in diverse colony types, we did not find a significant correlation between levels of C/EBPα and adipogenic potential in the single cell-derived colony assay. Results with viral-mediated dual expression of C/EBPα and bone morphogenetic protein (BMP)2 in C2C12 myoblasts suggest that C/EBPα may bias BMP2-induced osteoblasts towards adipogenesis [37]. In contrast, it is known that C/EBPβ/δ and Runx2 interact to enhance OCN transcription [38]. Taken together, the data indicate that these multiple transcription factors act together within carefully regulated levels to determine osteo-adipogenic lineage progression.

PPARs and their downstream targets (e.g., adipsin, [39]) are expressed during osteoblast development in our rat calvaria ObL model as well as two other independent in vitro osteoblastic models [17], [26]. However, mouse calvaria cells were reported to be more developmentally restricted than those multipotential mesenchymal cells expressing myoblast markers in addition to osteogenic or adipogenic markers, and were reported not to express PPARγ during osteogenic differentiation [17]. While our data are in general agreement, with detectable expression of several mesenchymal lineage determinants in rat calvaria ObL cells, we also detected PPARγ at both RNA and protein levels. However, our data suggest that the majority of the PPARγ, as well as Sox9 and MyoD, are transcriptionally inactive and have no functional consequences since they are abundant in the cytoplasm but not the nucleus throughout osteoblast differentiation. It is known that the PPARγ natural ligand 15-deoxy-Δ12,14-prostaglandin J2 (15d-PGJ2) induces nuclear translocation of PPARγ in mouse bone marrow stromal cells, whereas the reagent inhibits nuclear binding of Runx2 to DNA in the MC3T3-E1 and ST2 cells, in association with its adipogenic and anti-osteogenic activities [40]. Although the dynamics of nucleocytoplasmic shuttling of PPARγ in the presence of BRL are poorly understood, we found that BRL promoted the nuclear translocation of PPARγ in only a discrete subset of ObL cells and, indeed, Runx2/PPARγ-double nuclear-positive cells were occasionally observed. Likewise, Sox9, when activated, over-rides Runx2 function in ObL cells as shown by the phenotypes seen in transgenic mice in which Sox9 is driven by the type Iα collagen promoter [41]. Thus, activation of PPARγ (mostly γ2; see also below) may over-ride Runx2 function in collaboration with other adipogenic transcription factors, such as PPARα and C/EBPs under specific conditions, resulting in conversion of particular ObL cells into adipocytes, as shown here.

PPARγ has two major isoforms (γ1 and γ2), resulting from different promoter usage and alternative splicing. PPARγ2 is restricted predominantly to adipose tissue where it is crucial for adipogenesis, but the adipogenesis seen in PPARγ splice variant-null fibroblast cell lines suggests functional similarity between the isoforms [42]. Both PPARγ1 and γ2 are expressed in primary osteoblastic cells from human cancellous bone [43], while the mouse MC3T3-E1 cell line does not express PPARγ2 which led to the suggestion that PPARγ1 positively regulates osteoblastogenesis in this model [32]. Consistent with this view, osteogenic differentiation is correlated with upregulation of PPARγ1 (and the alternative transcripts γ3 and γ4) in two human osteoblast cell lines, SV-HFO and NHOST, and human mesenchymal stem cells [44]. Our observation that BRL enhanced nuclear translocation of PPARγ in ObL cells and increased CFU-ALP colonies begs the question of why bone nodule formation is maintained despite increased adipogenesis in ObL cultures treated with BRL. Our data suggest that PPARγ2 may have adipogenic and anti-osteogenic potential, while PPARγ1 may stimulate osteoblastogenesis, a possibility supported by results from microarray analysis of the mouse bone marrow cell line U-33 ectopically overexpressing PPARγ. In this genetically-engineered line, rosiglitazone up- and downregulates a large number of genes involved in multiple signaling pathways before the downregulation of the ObL determinants Runx2, Dlx5, Osterix [45]. Thus, PPARγ may be able to modulate osteoblast differentiation both dependently and independently of its negative effect on ObL determinants, and at multiple developmental stages.

In summary, we report that ObL cells co-express Runx2 and either PPARγ, Sox9, MyoD or a combination of regulatory factors for multiple mesenchymal lineages but while Runx2 translocates to the nucleus during osteogenic differentiation, the latter do not, rendering them inactive under osteogenic differentiation conditions. However, activation of PPARγ by treatment with its synthetic ligand, BRL, promotes nuclear translocation of PPARγ and induces an adipogenic fate switch in a discrete subset of ObL cells characterized by relatively high levels of endogenous PPARs. The molecular basis by which this subset of osteogenic cells acquires high endogenous expression of adipogenic transcription factors, whether regulated or occurring stochastically, remains to be determined.

## Materials and Methods

### Ethics Statement

Animal use and procedures were approved by the Institutional Animal Care and Use Committee at the Central Institute for Experimental Animals and the Committee of Animal Experimentation at Hiroshima University (#A09-36) and by the University of Toronto Animal Care Committee (#20008196).

### ObL cell culture

Cells were isolated from 21-day-old fetal Wistar rat calvariae by sequential collagenase (Type I; Sigma-Aldrich) digestion as described [46]. Cells from the last four fractions were separately grown in αMEM supplemented with 10% fetal bovine serum (Biological Industries) and antibiotics for 24 h. The cells were then trypsinized, pooled and grown at 0.35×10^4^ cells/cm^2^ in the same medium supplemented additionally with 50 µg/ml ascorbic acid (osteogenic medium). In particular experiments, cells were treated with or without BRL (≤100 nM) either in combination with or without RS (≤2.5%) or in combination with fenofibrate (≤100 nM), a synthetic ligand for PPARα.

### CFU assay

Cells were plated at 150 cells per well in 96-well plates in osteogenic medium with or without 1–100 nM BRL for 10 days and double-stained with the diazo method (CFU-ALP) and oil red O (CFU-adipocyte) (see below).

### Replica plating of single cell-derived colonies

Cells were plated at limiting dilution (≤15 cells/cm^2^ in 100-mm dishes) in osteogenic medium plus 10 nM dexamethosone (Dex), a stimulator of osteoblast differentiation in this model [47]. A few days later, polyester cloths (1 mm pore size) were placed over developing colonies for 24 h, then transferred upside down into new dishes with fresh osteogenic medium plus 10 nM Dex and 10 mM β-glycerophosphate (βGP) (replica dishes) [18], [24], [25]. Replica dishes were terminated at day 25 and subjected to ALP/von Kossa staining (see below). On days 12, 15, 17, and 21, colonies in master dishes were gently scraped from the dishes by using forceps and digested with trypsin and collagenase. The resulting cell suspension from each colony was split in half; one half was subjected to total RNA extraction and the other half was subcultured (∼2×10^4^/cm^2^) in osteogenic medium with 100 nM BRL. All cultures were maintained at 37°C in a humidified atmosphere with 5% CO_2_ and medium was changed every second or third day.

### Magnetic cell sorting

Differentiating cells in osteoid-like nodules (∼day10 of culture) were digested with collagenase and trypsin, and the resultant cell suspension was incubated with biotinylated anti-ALP antibody (40 µl/5×10^6^ cells; R&D systems, Minneapolis, MN) for 20 min at 4°C. After washing, the cells were labeled with anti-biotin magnetic microbeads (5 ml/5×10^6^ cells; Miltenyi Biotec) for 20 minutes at 4°C and applied onto a magnetic column (Miltenyi Biotec). After collection of the ALP pass-through fraction (ALP^−^), the column was removed from the magnetic field and the ALP^+^ fraction was flushed out according to the manufacturer's instructions. Control cells were prepared through the same process but with normal mouse IgG in place of anti-ALP antibody. An aliquot of each cell fraction (∼2.5×10^4^ cells/cm^2^) was replated and incubated in osteogenic medium with or without 100 nM BRL; the remainder of each fraction was used to measure ALP activity.

### ALP activity

Cells were washed with PBS and lysed by freeze-thawing (two times) in 0.05% TritonX 100. After centrifugation, ALP activity in cell lysates was measured with a LabAssay™ ALP assay kit (Wako Chemical) according to the manufacturer's instructions.

### ALP/von Kossa/oil red O staining

Cells were rinsed with PBS and fixed in 10% neutral buffered formalin. For ALP staining, the cells were incubated with naphthol AS MX/red violet or blue in 0.1M Tris-HCl (pH 8.3) as described elsewhere [46]. Matrix mineralization was confirmed by further incubation with 2.5% silver nitrate solution. For adipocytes, fixed or ALP-stained cells were air-dried and incubated in oil red O [48].

### RT-PCR

Total RNA was isolated from cells with TRIzol reagent (Invitrogen). Two micrograms of total RNA was reverse-transcribed by ReverTra Ace (Toyobo) at 50°C for 40 min. The sequence of primer sets for rat C/EBPα and δ [49], [50], OPN, ALP, BSP, OCN, and ribosomal protein L32 (internal control) were described elsewhere [47]; rat Runx2, PPARγ (directed to sequences in the 3′ end of the common region of γ1 and γ2), PPARγ1, PPARγ2, PPARα, MyoD, Sox9, lipoprotein lipase (LPL) and adipsin were designed using Primer Picking (primer 3) (Table S2). qRT-PCR was carried out according to the manufacturer's instructions (LightCycler; Roche Diagnostics) by using a SYBR Green 1 kit.

### Adaptor ligation-mediated PCR

To determine gene expression in single cell-derived colonies with limited cell number, high-fidelity global mRNA amplification was performed (TALPAT, T7 RNA polymerase promoter-attached, adaptor ligation-mediated, and PCR amplification followed by *in vitro* T7-transcription) [51]. Amplified cRNA was then reverse-transcribed, and qPCR was performed as above.

### Immunofluoroscence microscopy

Cells on coverslips were washed with PBS, fixed with ice-cold acetone and air-dried. Cells were then pretreated with Dako^R^ Protein Block at room temperature (RT) for 1 h, followed by incubation with primary antibodies (Runx2, PPARγ, Sox9, and MyoD; 1∶50; Santa Cruz Biotechnology) at 4°C overnight. Cy™3- and/or Cy™2-conjugated secondary antibodies (1∶400; Jackson Immunoresearch Laboratories) were used at RT for 1 h. Each incubation step was followed by two washes with PBS (5 minutes each). As negative control, normal goat or rabbit IgG (Vector) replaced primary antibodies.

### Western blotting

Cells were lysed with 100 mM KCl, 1 mM EDTA, 0.5% Nonidet P-40, 1 mM phenylmethylsulfonylfluoride and complete protease inhibitor (Roche Diagnostics) in 50 mM Tris-HCl (pH 7.5). Subcellular fractionation was carried out with a Qproteome Cell Compartment kit (Qiagen). Aliquots of samples were subjected to SDS-PAGE on 10–15% gels under reducing conditions and electroblotted onto nitrocellulose membranes. The membranes were treated with primary antibodies as above (1∶500) at 4°C overnight. The membranes were then incubated with horseradish peroxidase-conjugated secondary antibody (1∶2,000, Santa Cruz Biotechnology), followed by chemiluminescence detection. Anti-β-actin antibody (1∶1,000, Santa Cruz Biotechnology) was used as control.

### Statistical Analysis

Unless otherwise specified, data from triplicate samples are expressed as the mean ± SD, and a minimum of two independent experiments were performed. Statistical differences were evaluated by analysis of variance (ANOVA) and post hoc Student's t-test.

## Supporting Information

Figure S1 Morphological changes in RC cell population cultures in osteogenic medium with or without BRL. Cells were chronically treated with or without 100 nM BRL. Phase-contrast microscopy shows images at multiple development stages. Upper panels, because there is no morphological difference between cells with and without BRL until cell condensation, typical images of cells at day 3 (d3) and d6 in the presence of BRL are shown. Middle and bottom panels, cells at d8 and d10, respectively, in the presence (+) and absence (−) of BRL. Bottom panels are three times higher magnifications of the upper and middle panels. Arrows indicate adipocytes.
(3.32 MB TIF)

Figure S2 Expression profiling of mesenchymal lineage determinants in RC cell total population cultures. Cells were cultured under osteogenic conditions. Total RNA was isolated at the times indicated. (A) mRNA levels of Runx2, PPARγ1, PPARγ2, Sox9 and MyoD. Osteoblast markers such as OPN, ALP and OCN were also determined as an index of the stage of osteoblast development. Data are shown as relative abundance with ribosomal protein L32 used as internal control. (I) Western blotting of Runx2 and PPARγ. Whole cell lysates were extracted from parallel cultures to those in (A). Aliquots of samples were subjected to SDS-PAGE, blotted onto membranes and probed with appropriate antibodies.
(1.48 MB TIF)

Figure S3 Gene expression profiling of MyoD and Sox9 in single cell-derived ObL colonies. Numbers in each column denote relative mRNA levels of MyoD (Myo) and Sox9 by qRT-PCR. Light blue and blue are defined as in Figure 2. Blank space, Undetectable. S, Stages; IC, Individual colonies; see definitions in Figure 2.
(2.62 MB TIF)

Table S1 Osteo-adipogenic potential of indivisual colonies in the presence of BRL. Repl, Colony types identified by replica (Repl) plating, i.e., osteoblast (+) or non-osteoblast lineage (−). Subc, Staining patterns/developmental outcome in colonies subcultured (Subc) in the presence of BRL. ID, Colony ID. O, Oil red O positive; A, ALP positive; O/A, Oil red O/ALP double positive in subcultures with BRL.
(0.16 MB DOC)

Table S2 Primer sequences for qRT-PCR.
(0.04 MB DOC)
