# Supplementary figures and images for: A Subset of Osteoblasts Expressing High Endogenous Levels of PPARγ Switches Fate to Adipocytes in the Rat Calvaria Cell Culture Model
Source: PLoS One. 2010 Jul 26;5(7):e11782. doi: 10.1371/journal.pone.0011782 (PMC2909914; doi:10.1371/journal.pone.0011782)

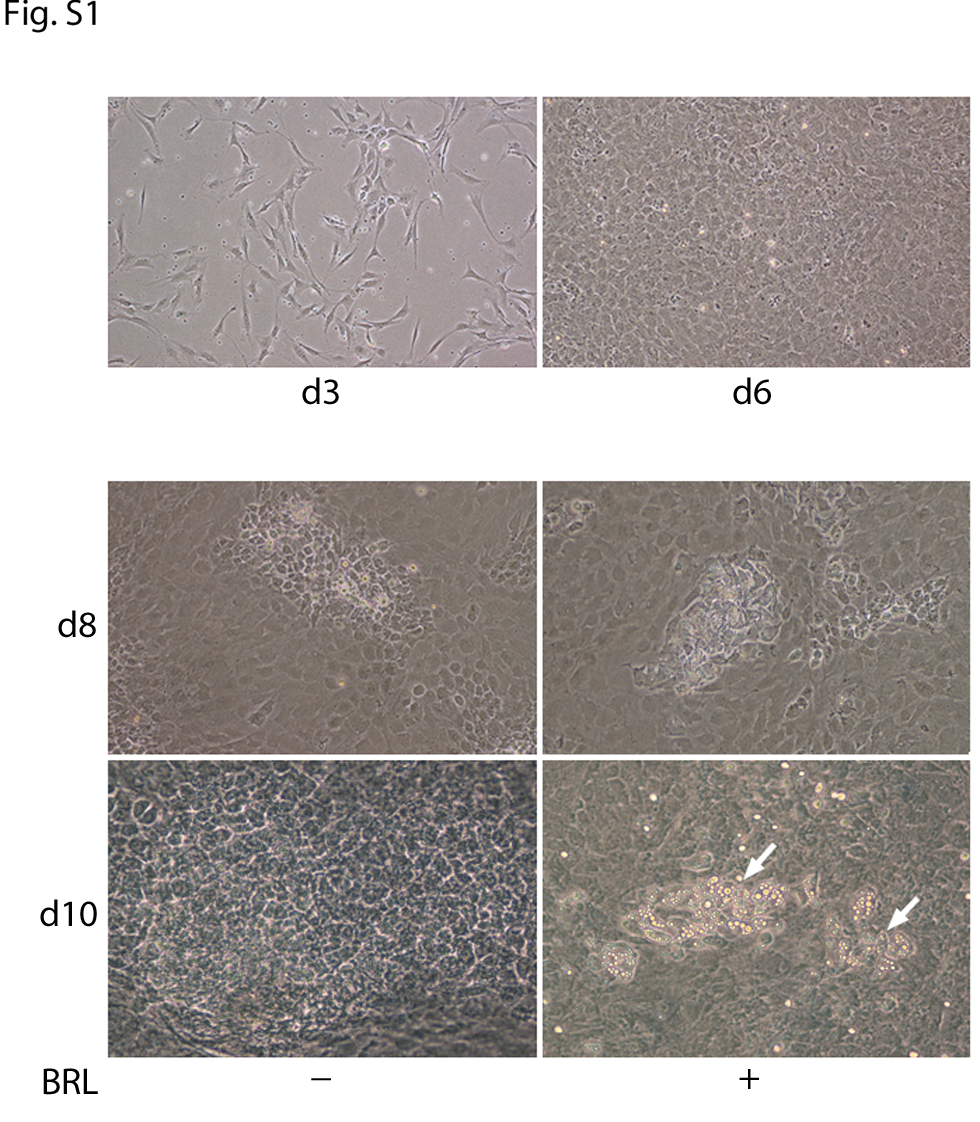

Supplement: Figure S1 — Morphological changes in RC cell population cultures in osteogenic medium with or without BRL. Cells were chronically treated with or without 100 nM BRL. Phase-contrast microscopy shows images at multiple development stages. Upper panels, because there is no morphological difference between cells with and without BRL until cell condensation, typical images of cells at day 3 (d3) and d6 in the presence of BRL are shown. Middle and bottom panels, cells at d8 and d10, respectively, in the presence (+) and absence (−) of BRL. Bottom panels are three times higher magnifications of the upper and middle panels. Arrows indicate adipocytes. (3.32 MB TIF) [file pone.0011782.s001.tif]

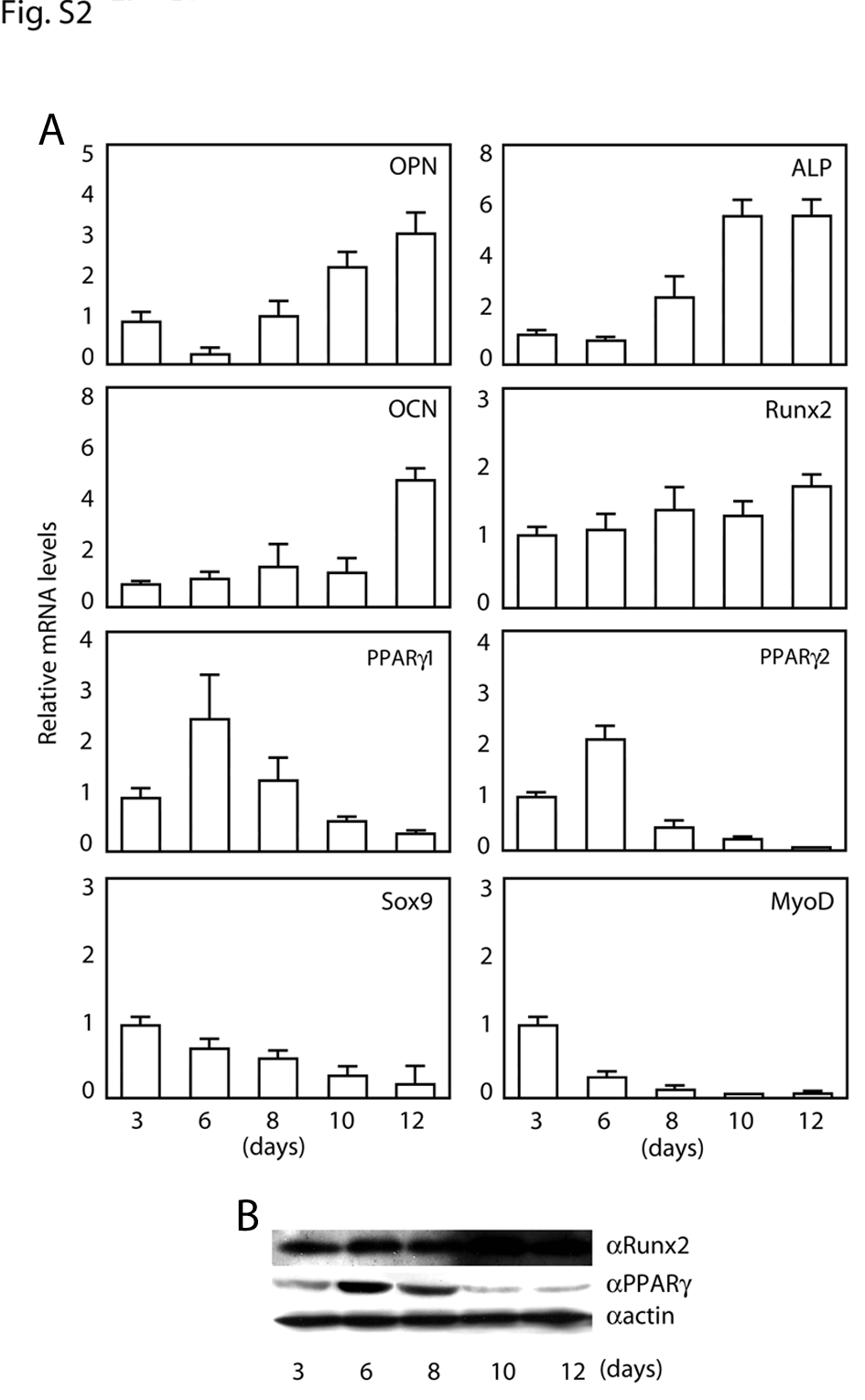

Supplement: Figure S2 — Expression profiling of mesenchymal lineage determinants in RC cell total population cultures. Cells were cultured under osteogenic conditions. Total RNA was isolated at the times indicated. (A) mRNA levels of Runx2, PPARγ1, PPARγ2, Sox9 and MyoD. Osteoblast markers such as OPN, ALP and OCN were also determined as an index of the stage of osteoblast development. Data are shown as relative abundance with ribosomal protein L32 used as internal control. (I) Western blotting of Runx2 and PPARγ. Whole cell lysates were extracted from parallel cultures to those in (A). Aliquots of samples were subjected to SDS-PAGE, blotted onto membranes and probed with appropriate antibodies. (1.48 MB TIF) [file pone.0011782.s002.tif]

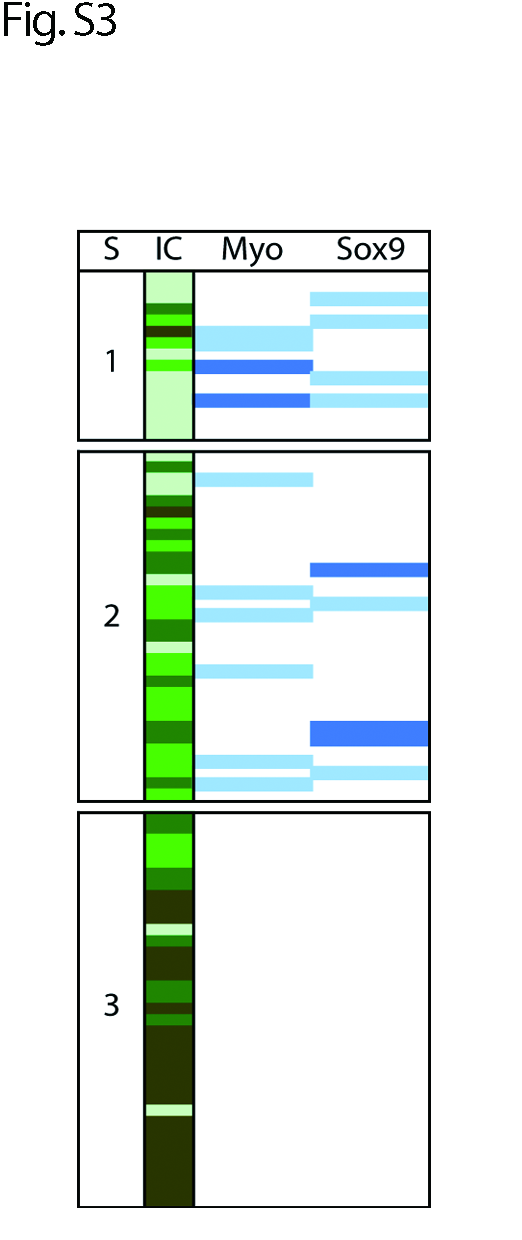

Supplement: Figure S3 — Gene expression profiling of MyoD and Sox9 in single cell-derived ObL colonies. Numbers in each column denote relative mRNA levels of MyoD (Myo) and Sox9 by qRT-PCR. Light blue and blue are defined as in Figure 2. Blank space, Undetectable. S, Stages; IC, Individual colonies; see definitions in Figure 2. (2.62 MB TIF) [file pone.0011782.s003.tif]
