# Supplementary material for: A Subset of Osteoblasts Expressing High Endogenous Levels of PPARγ Switches Fate to Adipocytes in the Rat Calvaria Cell Culture Model
Source: PLoS One. 2010 Jul 26;5(7):e11782. doi: 10.1371/journal.pone.0011782 (PMC2909914; doi:10.1371/journal.pone.0011782)
Supplement: Table S1 — Osteo-adipogenic potential of indivisual colonies in the presence of BRL. Repl, Colony types identified by replica (Repl) plating, i.e., osteoblast (+) or non-osteoblast lineage (−). Subc, Staining patterns/developmental outcome in colonies subcultured (Subc) in the presence of BRL. ID, Colony ID. O, Oil red O positive; A, ALP positive; O/A, Oil red O/ALP double positive in subcultures with BRL. (0.16 MB DOC) [file pone.0011782.s004.doc]

| Colony | | | |  | Colony | | | |  | Colony | | | |  | Colony | | | |
| --- | --- | --- | --- | --- | --- | --- | --- | --- | --- | --- | --- | --- | --- | --- | --- | --- | --- | --- |
| Day | ID | Repl | Subc |  | Day | ID | Repl | Subc |  | Day | ID | Repl | Subc |  | Day | ID | Repl | Subc |
| 12 | 2 | - |  |  | 15 | 1 | + | O/A |  | 17 | 3 | + |  |  | 21 | 1 | + | O/A |
|  | 5 | - | O |  |  | 3 | + |  |  |  | 5 | + | O/A |  |  | 3 | + | A |
|  | 6 | + | O |  |  | 4 | + |  |  |  | 7 | + | O/A |  |  | 5 | - |  |
|  | 7 | + | O |  |  | 5 | + | O/A |  |  | 8 | + | O/A |  |  | 6 | + | A |
|  | 13 | - |  |  |  | 6 | + | O/A |  |  | 10 | + | O |  |  | 8 | + | A |
|  | 15 | + | O |  |  | 10 | + | O |  |  | 12 | - |  |  |  | 10 | + | A |
|  | 16 | - |  |  |  | 12 | + | O |  |  | 14 | - |  |  |  | 11 | + | O/A |
|  | 17 | - |  |  |  | 13 | + |  |  |  | 15 | + |  |  |  | 13 | + |  |
|  | 20 | + |  |  |  | 14 | + | O/A |  |  | 16 | + |  |  |  | 14 | + |  |
|  | 21 | + | O |  |  | 16 | + | O/A |  |  | 19 | - |  |  |  | 16 | + |  |
|  | 22 | + | O |  |  | 18 | + | O |  |  | 20 | + | O/A |  |  | 18 | + |  |
|  | 24 | + | O |  |  | 21 | + |  |  |  | 22 | + | A |  |  | 19 | + | A |
|  | 26 | + |  |  |  | 22 | + | O/A |  |  | 24 | + | O/A |  |  | 22 | + |  |
|  | 27 | - | O |  |  | 24 | - |  |  |  | 25 | - |  |  |  | 24 | + | A |
|  | 31 | - |  |  |  | 26 | + | O/A |  |  | 27 | + |  |  |  | 25 | + | A |
|  | 32 | + | O/A |  |  | 27 | + | O/A |  |  | 29 | + | A |  |  | 28 | + | A |
|  | 33 | + | O |  |  | 28 | + | O/A |  |  | 30 | + |  |  |  | 29 | - |  |
|  | 34 | - |  |  |  | 29 | - |  |  |  | 33 | + | O/A |  |  | 32 | + |  |
|  | 38 | - |  |  |  | 32 | + | O/A |  |  | 36 | - |  |  |  | 33 | + | A |
|  | 39 | - |  |  |  | 33 | + |  |  |  | 37 | + |  |  |  | 35 | + | A |
|  | 40 | + | O |  |  | 34 | + |  |  |  | 40 | + |  |  |  | 36 | + | A |
|  | 42 | + | O |  |  | 36 | + |  |  |  | 42 | + | A |  |  | 38 | + | A |
|  | 43 | + | O |  |  | 37 | + | O/A |  |  | 44 | + | A |  |  | 40 | + | A |
|  | 45 | + | O |  |  | 39 | + | A |  |  | 45 | + |  |  |  | 42 | + |  |
|  | 47 | + |  |  |  | 41 | + | O/A |  |  | 46 | + | A |  |  | 44 | + |  |
|  | 49 | + |  |  |  | 42 | + |  |  |  | 48 | + | A |  |  | 45 | + |  |
|  | 50 | - |  |  |  | 44 | + |  |  |  | 49 | + |  |  |  | 47 | + | A |
|  | 53 | + | O |  |  | 45 | + |  |  |  | 51 | + |  |  |  | 48 | + |  |
|  | 55 | - |  |  |  | 47 | + | A |  |  | 55 | + | O/A |  |  | 49 | + |  |
|  | 56 | + | O |  |  | 48 | - |  |  |  | 56 | + | A |  |  | 52 | + | O/A |
|  | 57 | + | A |  |  | 50 | - |  |  |  | 57 | - |  |  |  | 53 | + | A |
|  | 60 | - |  |  |  | 51 | + | A |  |  | 59 | + |  |  |  | 56 | + | A |
|  | 61 | + |  |  |  | 53 | + | O/A |  |  | 60 | + |  |  |  | 58 | + | A |
|  | 62 | + |  |  |  | 55 | + | O/A |  |  | 62 | + | O/A |  |  | 59 | + |  |
|  | 64 | + | O/A |  |  |  |  |  |  |  | 64 | + |  |  |  | 61 | + | A |
|  | 65 | + | O |  |  |  |  |  |  |  | 65 | + | O/A |  |  | 63 | + | A |
|  |  |  |  |  |  |  |  |  |  |  | 66 | + | O |  |  | 65 | + |  |
|  |  |  |  |  |  |  |  |  |  |  | 68 | + |  |  |  | 67 | + | A |
|  |  |  |  |  |  |  |  |  |  |  | 70 | + | O/A |  |  | 69 | + |  |
|  |  |  |  |  |  |  |  |  |  |  | 72 | - |  |  |  | 73 | + | A |
|  |  |  |  |  |  |  |  |  |  |  | 73 | - |  |  |  | 77 | + |  |
|  |  |  |  |  |  |  |  |  |  |  | 74 | + | O/A |  |  |  |  |  |
