# Supplementary material for: A Subset of Osteoblasts Expressing High Endogenous Levels of PPARγ Switches Fate to Adipocytes in the Rat Calvaria Cell Culture Model
Source: PLoS One. 2010 Jul 26;5(7):e11782. doi: 10.1371/journal.pone.0011782 (PMC2909914; doi:10.1371/journal.pone.0011782)
Supplement: Table S2 — Primer sequences for qRT-PCR. (0.04 MB DOC) [file pone.0011782.s005.doc]

|  | C/EBPα | 5’-AGTTGACCAGTGACAATGACCG-3’ and 5’-TCAGGCAGCTGGCGGAAGAT-3’ |  |
| --- | --- | --- | --- |
|  | C/EBPδ | 5’-CGAGGTGACAGCCCAACTTG-3’ and 5’-TCGGTCGTTCGGAGTCTCTAA G-3’ |  |
|  | OPN | 5’-AGAGGAGAAGGCGCATTACA-3’ and 5’-GCAACTGGGATGACCTTGAT-3’ |  |
|  | ALP | 5’-TTAAGGGCCAGCTACACCAC-3’ and 5’-GATAGGCGATGTCCTTGCAG-3’ |  |
|  | BSP | 5’-CGCCTACTTTTATCCTCCTCTG-3’ and 5’-CTGACCCTCGTAGCCTTCATAG-3’ |  |
|  | OCN | 5’-AGGACCCTCTCTCTGCTCAC-3’ and 5’-AACGGTGGTGCCATAGATGC-3’ |  |
|  | PPARγ | 5’-GTCAGCGACTGGGACTTTTC-3’ and 5’-CGAGGACATCCAAGACAACC-3’ |  |
|  | PPARγ1 | 5’-TAAGGGACTCGAGGAGGT CA-3’ and 5’-GCTCTTGTGAACGGGATGTCA-3’ |  |
|  | PPARγ2 | 5’-TGGGAGATCCTCCTGTTGAC-3’ and 5’-CCATAGTGGAAGCCTGATGC-3’ |  |
|  | PPARα | 5’-CGACAAGTGTGATCGAAGCTGCAAG-3’ and 5’-GTTGAAGTTCTTCAGGTAGGCTTC-3’ | |
|  | MyoD | 5’-AGGGAAGGGAAGAGCAGAAG-3’ and 5’-CTGTGGGAAAGAGTGGGTGT-3’ |  |
|  | Sox9 | 5’-CTGAAGAAGAAGGAGAGCGAGGA-3’ and 5’-CGGGGCTGGTACTTGTAATC-3’ |  |
|  | LPL | 5’-GAGATTTCTCTGTATGGCACA-3’ and 5’-CTGCAGATGAGAGAAACTTTCTC-3’ |  |
|  | Adipsin | 5’-TGT ACT TCG TGG CTC TGG TG-3’ and 5’-TGTACTTCGTGGCTCTGGTG-2’ |  |
|  | L32 | 5’-CATGGCTGCCCTTCGGCCTC-3’ and 5’-CATTCTCTTCGCTGCGTAGCC-3’ |  |
